# Metal Nanomaterials: A Strategy to Combat Drug-Resistant Bacterial Infections

**DOI:** 10.34133/research.0853

**Published:** 2025-08-21

**Authors:** Yaran Wang, Fan Wu, Yijin Ren, Yong Liu, Henny C. Mei

**Affiliations:** ^1^ State Key Laboratory of Medicinal Chemical Biology, Key Laboratory of Functional Polymer Materials, Ministry of Education, Institute of Polymer Chemistry, College of Chemistry, Nankai University, Tianjin 300071, China.; ^2^ Department of Biomaterials and Biomedical Technology, University of Groningen and University Medical Center Groningen, Groningen, Netherlands.; ^3^ Department of Orthodontics, University of Groningen and University Medical Center Groningen, Groningen, Netherlands.

## Abstract

Bacterial infections pose a major challenge today due to the rise of drug resistance in pathogenic bacteria, creating an urgent need for the development of nonantibiotic therapies. Metal nanomaterials are increasingly recognized as promising antibacterial agents due to their unique physical and chemical properties, which offer strong antibacterial capabilities and broad-spectrum activity against drug-resistant bacteria. Unlike traditional antibacterial therapies, metal nanomaterials rarely induce drug resistance because of their diverse antibacterial mechanisms, which destroy bacteria by directly damaging bacterial cells or generating oxidative stress. Metal nanomaterials have proven effective in treating various drug-resistant bacterial infections. This perspective highlights recent advances in metal nanomaterials for antimicrobial applications. Firstly, bacterial infections and the current dilemma are introduced. Next, recent progress in the antibacterial activity of metal nanomaterials against drug-resistant bacteria is summarized, along with the challenges in their antimicrobial applications. Finally, future prospects and remaining challenges associated with the use of metal nanomaterials to treat drug-resistant bacteria are discussed.

## Bacterial Infections: The Current Antibiotic Dilemma

Bacterial infections have emerged as a global challenge and a major threat to human health. Since the discovery of antibiotics, previously fatal bacterial infections have been largely controlled. However, excessive antibiotic use and microbial evolution have led to antibiotic resistance [1,2]. Microorganisms acquire resistance mainly through genetic mutations that alter antibiotic binding sites, reducing target affinity and decreasing effectiveness. Additionally, resistance mechanisms such as enzymatic inactivation, efflux pumps, alternative metabolic pathways, and altered permeability have further reduced antibiotic efficacy [3,4]. Biofilm formation worsens this problem, as bacteria in biofilms are protected from antibiotics and the immune system by the extracellular matrix. This makes it hard for drugs to penetrate and kill the bacteria [5]. As a result, antibiotic therapy has become less effective against drug-resistant infections. There is now an urgent need for novel, effective nonantibiotic strategies to combat drug-resistant bacteria.

## Antibacterial Activity of Metal Nanomaterials

Nanomaterials are considered a promising alternative to antibiotics due to their low cost, convenient synthesis methods, biocompatibility, environmental friendliness, and broad applicability [6]. Among these, metal nanomaterials are currently one of the fastest developing and most widely used, owing to their long history in antibacterial applications, such as the traditional use of gold and silverware before the advent of antibiotics [7]. Their nanoscale size and high surface-to-volume ratio enable superior physical and chemical properties and antibacterial activity compared to bulk metal materials. To date, metal nanomaterials have been developed in various formulations, including pure metals, alloys, metal oxides, metal sulfides, and metal-organic frameworks, with applications in drug delivery and antimicrobial therapy.

**Figure 1.**
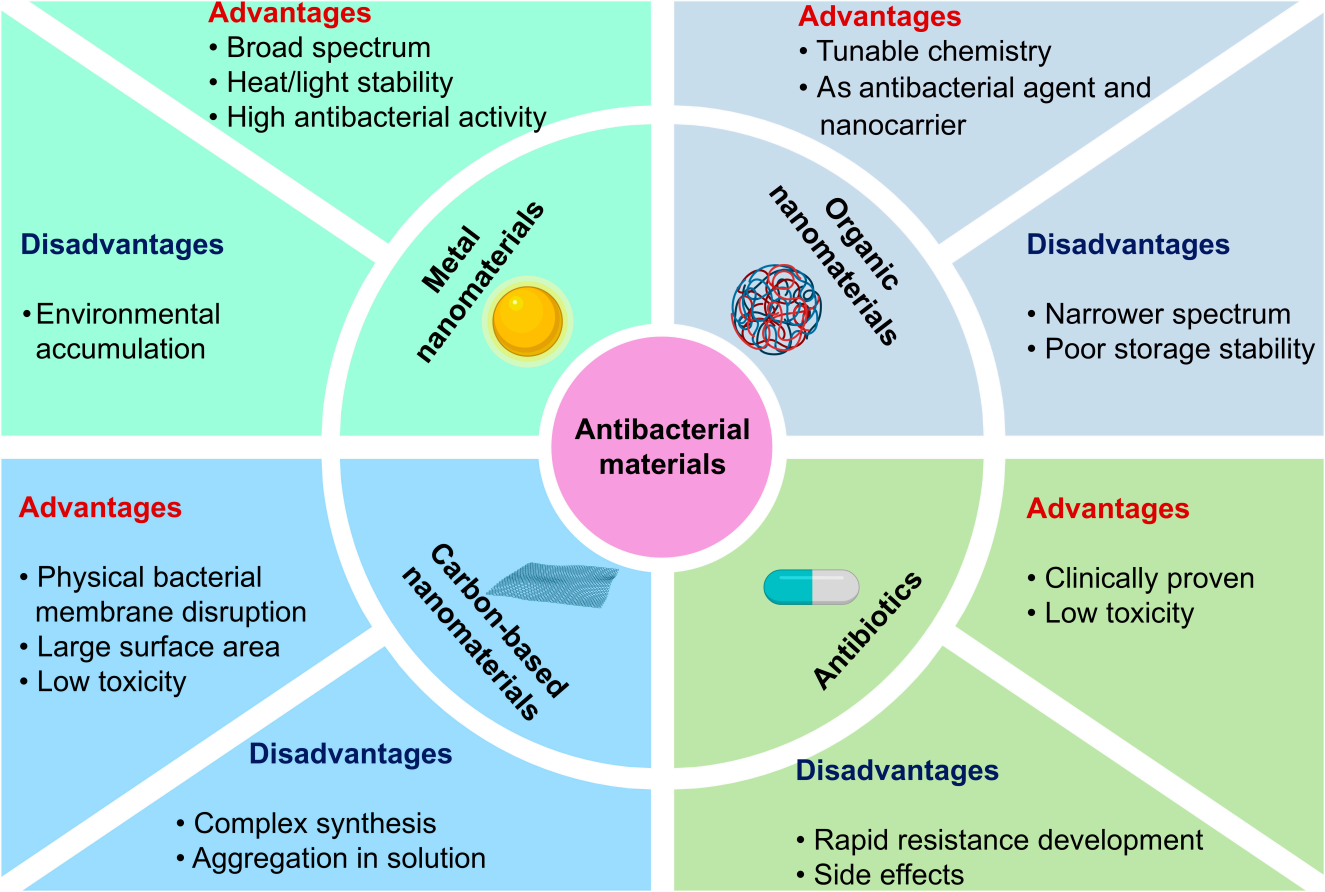
Comparison of metal nanomaterials with antibiotics, organic nanomaterials, and carbon-based nanomaterials.

Unlike traditional antibiotics that act on specific molecular targets (a key driver of antimicrobial resistance), metal nanomaterials exert multifaceted, nonspecific antibacterial mechanisms, such as membrane disruption and reactive oxygen species (ROS) generation [8–10]. These nontargeted modes of action reduce the potential for resistance development and provide broader-spectrum antimicrobial activity compared to antibiotics (Figure 1) [11]. Other nanomaterials like carbon-based and organic nanomaterials such as graphene oxide are interacting and disrupting cell membranes and can produce ROS with photodynamics but have a disadvantage: they have only a few mechanisms to kill multidrug-resistant bacteria.

Furthermore, metal nanomaterials demonstrate high efficacy against biofilms due to their nonspecific targeting and penetration capabilities. Table 1 provides a comprehensive overview of various metal nanomaterials, highlighting their different antibacterial mechanism to overcome bacterial infections. One of the primary mechanisms involves ROS generation, in which metal nanomaterials such as Ag nanoparticles and ZnO nanoparticles induce oxidative stress and cause bacterial cell damage. This mechanism is especially effective against drug-resistant bacteria, as ROS can target various bacterial biomolecules, thereby reducing the likelihood of resistance development [12]. Moreover, metal nanomaterials are capable of bypassing bacterial resistance mechanisms through modulation of bacterial membrane efflux and permeability, release of heavy metal ions, excitation of phototherapy, and effective prevention of biofilm formation, thereby enhancing their antimicrobial efficacy [13]. This makes them promising candidates in the ongoing battle against drug-resistant pathogens.

**Table 1.** A comprehensive overview of various metal nanomaterials for antibacterial applications

Nanomaterials	Antibacterial mechanisms	Advantages	Disadvantages	Combination strategies	Infection models used in experimental animals	Clinical trial ^a^
Au-based	• Inhibit intracellular ATP synthesis and tRNA binding • Induce oxidative stress • Increase cellular membranes permeability	• Easy to synthesize through physical, chemical, or biological methods • Easy-tunable surface properties, size, and shape (e.g., spheres, tubes, or flowers) • Photothermal capability • Excellent biosafety	• Narrow spectrum of activity • Poor storage stability • High production costs	• Photothermal therapy • Glucose oxidase	• Peritonitis infection • Wound healing • Oral infection	Yes
Ag-based	• Release Ag^+^ to damage cell membrane and DNA • Inhibit efflux pump • Depolarize cellular membrane • Generate ROS to damage DNA and proteins	• Easy to synthesize through physical, chemical, or biological methods • Controllable size and morphology • Diverse formulations (e.g., coating or emulsion) • Synergistic effects with antibiotics	• Mechanism of biofilm penetration and killing not fully understood • High production costs • Potential toxicity	• Photothermal therapy • Combination with antibiotics	• Wound healing • Urinary tract infection • Bacterial diarrhea	Yes
Cu-based	• Release copper ions to damage cell membrane and DNA • Generate ROS to damage DNA and proteins	• Chemically and physically stable • Cost-effective • Broad-spectrum activity • Low cytotoxic side effects	• Difficult dosage control • Low dispersibility	• Photothermal therapy • Glucose oxidase and peroxidase	• Wound healing • Sepsis • Bacterial vaginosis	Yes
Zn-based	• Release zinc ions to damage cell membrane and DNA • Generate ROS to damage DNA and proteins	• Cost-effective • Broad-spectrum activity	• Potential cytotoxic • Limited antibacterial activity	• Photodynamic therapy	• Wound healing • Periodontitis	Yes
Ti-based	• Generate ROS with light irradiation	• Excellent stability • Nontoxic • Ideal for implant surface modifications	• UV light needed • Limited antibacterial activity	• Photodynamic therapy	• Subcutaneous infection • Wound infection • Oral infection	Yes
Fe-based	• Generate ROS via Fenton reaction	• Controllable size and morphology • Magnetic targeting potential • Low cost	• Narrow effective pH range • Potential toxicity	• Peroxidase • Photothermal therapy	• Wound infection • Oral infection	No
Ce-based	• Promotes ROS generation via redox cycle (Ce^3+^/Ce^4+^)	• Self-regulating redox state • Prolonged antibacterial action	• Unstable	• Peroxidase • Oxidase	• Wound infection	No
Pd-based	• Physical interaction with cell membrane, creating pores • Inactivation of cellular enzymes	• Photothermal properties • Hydrogen storage	• Biocompatibility concerns • Uncertain biodistribution	• Hydrogen therapy • Photothermal therapy	• Wound infection • Oral infection	No

^a^ Clinical trial data were retrieved from https://clinicaltrials.gov/

## Challenges in Antibacterial Applications of Metal Nanomaterials

The evolution of bacterial resistance to metal nanomaterials is still in its early stages. Effective resistance prevention requires multiple antibacterial mechanisms. Emerging evidence suggests that prolonged use of metal nanomaterials may induce resistance. For instance, *Escherichia coli* and *Pseudomonas aeruginosa* treated with Ag nanoparticles have exhibited resistance through phenotypic changes, including enhanced flagellar assembly that promotes Ag aggregation [14]. Therefore, synergistic approaches combining metal nanomaterials with other antibacterial strategies, such as photothermal therapy, chemodynamic therapy, or photodynamic therapies, hold great promise. These approaches offer enhanced efficacy while reducing resistance risks.

The biosafety of metal nanomaterials represents a major barrier to clinical translation. Unlike traditional medicines, they may induce oxidative stress, inflammation, and cellular damage [15,16]. Their cytotoxicity strongly depends on physicochemical properties, including size, shape, surface charge, composition, and solubility. For instance, positively charged metal nanomaterials penetrate cell membranes more readily and interact with intracellular organelles than negatively charged ones [17]. Several methods have been employed to improve biosafety, including biosynthesis, surface modification with natural compounds, and hybridization with other nanomaterials to reduce metal concentrations while maintaining activity [18]. It is important to avoid the accumulation of metal nanomaterials in the human body; therefore, biodegradation and metabolism also need to be studied. The distribution and clearance of metal nanomaterials can be tracked by labeling with radiotracers in animal and human studies. Furthermore, the presence of metal ions in biological samples (e.g., blood, urine, and tissues) needs to be monitored.

Most applications of metal nanomaterials remain preclinical, with animal studies dominating current research. In vivo evaluations using various infection models, ranging from wound healing and oral infections to systemic conditions like peritonitis, have provided valuable therapeutic insights [19]. While some metal nanomaterials, including Au-, Ag-, Cu-, Zn-, Ti-, and Fe-based nanomaterials, have been used in clinical trials, their current applications focus primarily on diagnostics rather than infection treatment. For example, Au (NCT04907422) is being used as a biomarker, Fe (NCT00920023) is used for tumor imaging, and Cu (NCT04334837) is used for tumor detection. These examples demonstrate their clinical potential while emphasizing the need for further optimization and comprehensive clinical evaluation to advance infection applications from laboratory research to clinical trials and ultimately to market approval. The translational imperative hinges on establishing robust preclinical validation and iterative clinical feedback. Firstly, a thorough evaluation of the safety profiles of metal nanomaterials in vivo is essential, including prolonged toxicity, biodistribution, and metabolic pathways. Then, extensive clinical datasets derived from diverse human populations are essential. This involves integrating rigorous preclinical validation with human data feedback, driven by synergistic partnerships between academia, industry, and the regulatory sector.

## Conclusions and Future Prospects

Over the past 5 years, nearly 9,000 papers (according to Web of Science, keywords: “metal nanomaterial” and “antibacterial”; assessment date: May 2025) have been published on metal nanomaterials for antibacterial infections. However, the rapid growth in research has far outpaced practical applications, with most advancements remaining confined to laboratory settings. Therefore, further efforts should prioritize multidisciplinary in-depth research, including studies on drug-resistance mechanisms, long-term biosafety, and clinical trials, rather than on developing more new metal nanomaterials. To effectively prevent antibacterial resistance, the underlying mechanism must be fully understood through comprehensive long-term in vitro and in vivo studies. Since most bacterial infections involve multiple species, in vitro studies should use mixed species biofilms to better reflect clinical conditions. Finally, for successful clinical translation, the production process must meet 3 key criteria: high quality standards, scalability, and cost-effectiveness.
